# Impact of Parakinesia brachialis oscitans on limb functional recovery after stroke: a cohort study

**DOI:** 10.3389/fneur.2026.1824166

**Published:** 2026-05-08

**Authors:** Congcong Wang, Hua Hu, Runying Wang, Bin Xu, Jia Du, Zhou Su, Shuangxi Guo, Jingkai Wang, Xiaojun Tian

**Affiliations:** 1 Department of Neurology, Henan Medical Key Laboratory of Neurology, Henan Joint International Laboratory of Neurorestoratology for Senile Dementia, Henan Key Laboratory of Neurorestoratology and Protein Modification, The First Affiliated Hospital of Henan Medical University, Xinxiang, Henan, China; 2 College of Pharmacy, Heilongjiang University of Chinese Medicine, Harbin, Heilongjiang, China; 3 Department of Neurosurgery, Henan Medical University, Zhengzhou, China; 4 Zhengzhou Central Hospital Affiliated to Zhengzhou University, Zhengzhou, China

**Keywords:** Fugl-Meyer assessment (FMA), motor ability, Parakinesia brachialis oscitans, propensity score matching, stroke

## Abstract

**Background:**

Parakinesia brachialis oscitans (PBO), involuntary elevation of the paralyzed upper limb during yawning is a rare phenomenon after ischemic stroke, but its prognostic significance remains unclear due to limited systematic studies.

**Objective:**

To compare 3-month motor recovery between ischemic stroke patients with PBO, without PBO, and with spontaneously resolved PBO using a propensity score-matched design.

**Methods:**

This retrospective cohort study included 33 patients with PBO identified in the database as the PBO group, while the non-PBO group was selected from patients with acute ischemic stroke between March and June 2024. Two independent neurologists reviewed medical records according to standardized criteria to identify PBO (within 7 days of stroke onset) and collected baseline data from all patients within 24 h of stroke onset. PSM (1:2) was used to balance baseline characteristics (age, sex, vascular risk factors, stroke location, ASPECTS score, baseline Lovett score, and FMA score) between the PBO and non-PBO groups. The primary outcome was the FMA score at 3 months; the secondary outcome was the Lovett classification. All outcome assessors were blinded.

**Results:**

After matching, 26 PBO patients were matched to 52 non-PBO patients with balanced baseline characteristics. At 3 months, the PBO group showed significantly higher FMA scores (mean difference 3.77, 95% CI: −0.54–6.97; *p* = 0.022) and Lovett grades (*p* = 0.018) than the non-PBO group. Among PBO patients, the PBO disappearance group (*n* = 19) within 1 month achieved higher FMA scores than the PBO persistence group (*n* = 14) (mean difference 7.11, 95% CI: −13.10–-1.06; *p* = 0.021), whereas Lovett grades did not differ significantly (*p* = 0.111).

**Conclusion:**

In this propensity-score-matched cohort study, the presence of PBO was associated with better recovery of motor function at 3 months. Early spontaneous resolution of PBO was associated with better fine motor outcomes compared to persistent PBO. These findings, which support the hypothesis, suggest that PBO may serve as a potential prognostic indicator warranting further prospective research.

## 1 Introduction

Ischemic stroke is the most common disabling disease in China. With the acceleration of population aging, its incidence continues to rise, with over 3 million new cases annually (1). The resulting loss of working capacity imposes significant life and economic burdens on patients and their families. Early rehabilitation therapy, particularly limb function rehabilitation, significantly impacts patients’ subsequent quality of life (2). 70 to 80% of patients experience varying degrees of motor impairment, and 50 to 60% of patients continue to have functional impairments 6 months later (3).

Parakinesia brachialis oscitans (PBO) refers to the phenomenon where paralyzed limbs exhibit involuntary movement during yawning, observed after neurological damage causes loss of voluntary limb movement. This uncommon occurrence was first termed PBO by Walusinski et al. (4). Current research on PBO, both domestically and internationally, remains limited to scattered case reports and mechanistic hypotheses. Systematic, multimodal explanations of its mechanisms are lacking, and high-level evidence-based medical evidence linking PBO to rehabilitation prognosis is absent (5). Clinically, rehabilitation outcomes for patients exhibiting PBO appear distinct from those without this phenomenon. A previous study indicated that 25% of patients with PBO remained paralyzed, while the others regained varying degrees of limb mobility; only 5% regained hand function (6). The PBO phenomenon may serve as a clinical biomarker capable of predicting a patient’s potential for functional recovery, guiding the timing and intensity of rehabilitation interventions to achieve better functional outcomes. Against this backdrop, we conducted a cohort study to investigate whether the presence of PBO, as well as its persistence versus resolution within the first month post-stroke, is associated with differences in functional recovery (assessed at 3 months) in patients with ischemic stroke.

## 2 Methods

### 2.1 Population

This was a retrospective cohort study using data from a stroke registry at the First Affiliated Hospital of Henan Medical University. Patients with clear manifestations of PBO from January 2022 to March 2024 were selected from the database to form the study group, comprising a total of 33 cases, designated as the PBO group. Because PBO is an intermittent condition, case confirmation is based on cross-validation of video evidence, clinical records, and nursing notes. Each PBO case in the registry includes at least one recorded video clip. Two independent neurologists (Bin Xu and Jia Du) independently review the videos and clinical records according to predefined diagnostic criteria: (1) involuntary elevation of the paralyzed upper limb during yawning; (2) return of the limb to its pre-yawn position upon completion of the yawn; and (3) exclusion of involuntary movements caused by other conditions (e.g., epilepsy and myoclonus). When discrepancies arise, the cases are reviewed jointly until a consensus is reached. The time of onset is defined based on the earliest recorded event in the medical or nursing records. Patients diagnosed with and treated for ischemic stroke at our hospital between March and June 2024 were selected. Inclusion criteria: ① Age 55–75 years; ② Meeting diagnostic and treatment criteria outlined in the 2021 AHA/ASA Guidelines for the Diagnosis and Treatment of Acute Ischemic Stroke (7); ③ Confirmed acute cerebral infarction via cranial MRI-DWI. ④ Informed consent obtained from patients. Exclusion Criteria: ① Severe psychiatric disorders or cognitive impairment preventing follow-up compliance; ② Unstable cardiovascular disease or poorly controlled chronic conditions; ③ Concurrent fractures or skeletal muscle injuries impairing motor function. Dropout Criteria: ① Patient or family refusal to participate; ② Development of new conditions affecting motor function during the study period, such as fractures. A total of 636 patients meeting these criteria were screened. Baseline characteristics were described for the 33 PBO patients and 636 patients without PBO manifestations. All patients received standardized rehabilitation management. This information was extracted from electronic medical records and rehabilitation logs.

## 3 Data collection

### 3.1 Baseline data collection

PBO occurrence was documented within the first 7 days post-stroke. Baseline functional assessments were performed at the time of hospital admission (within 24 h post-stroke). Recorded for all subjects: gender, age, stroke location (anterior/posterior circulation), hypertension, diabetes, homocysteine (HCY), low-density lipoprotein (LDL). Early ischemic changes in the anterior circulation were quantitatively assessed using the Alberta Stroke Program Early CT Score (ASPECTS) (8). For posterior circulation (vertebrobasilar artery system) infarction, evaluation was performed using the posterior circulation ASPECTS (pC-ASPECTS) (9).

### 3.2 The timing of PBO onset was recorded relative to the baseline assessment

For patients with PBO, the duration of the phenomenon was defined as the interval between the first documented occurrence and the last observed episode. Patients were classified into the disappearance group if PBO resolved within 1 month post-stroke and the persistence group if PBO continued beyond 1 month.

### 3.3 Lovett muscle strength grading scale

Immediately upon admission and at 3 months, the Lovett muscle strength grading scale was used to assess muscle strength in the upper and lower limbs affected by the current stroke event. Muscle strength was categorized into grades 0 to 5: Grade 0: No observable muscle contraction; Grade 1: Slight muscle contraction but unable to produce joint movement. Grade 2: Poor muscle strength, i.e., able to perform a full range of motion at the horizontal plane. Grade 3: Fair strength, allowing full range of motion against gravity but not against resistance. Grade 4: Good strength, allowing movement against moderate resistance. Grade 5: Normal strength, allowing movement against gravity and sufficient resistance.

### 3.4 The Fugl-Meyer assessment scale (FMA)

The Fugl-Meyer Assessment Scale (FMA) was assessed immediately after stroke and 3 months post-stroke. This scale comprises two sections: upper limb and lower limb. The upper limb section assesses four domains:arm, wrist, hand, and coordination. Each item is scored from 0 to 2 points, yielding a maximum total of 66 points. The lower limb section comprises three domains: supine position, sitting position, and standing position. Each item is scored from 0 to 2 points, with a maximum score of 34 points. Higher scores indicate better motor function (10). The Lovett score and FMA score for the PBO group were obtained from the database, while the data for the non-PBO group were derived from follow-up records. Primary outcome measure was defined as the FMA score at 3 months, reflecting upper and lower limb motor function. Secondary outcome measures included the Lovett score at 3 months, reflecting muscle strength.

### 3.5 Propensity score matching (PSM)

**Figure 1:**
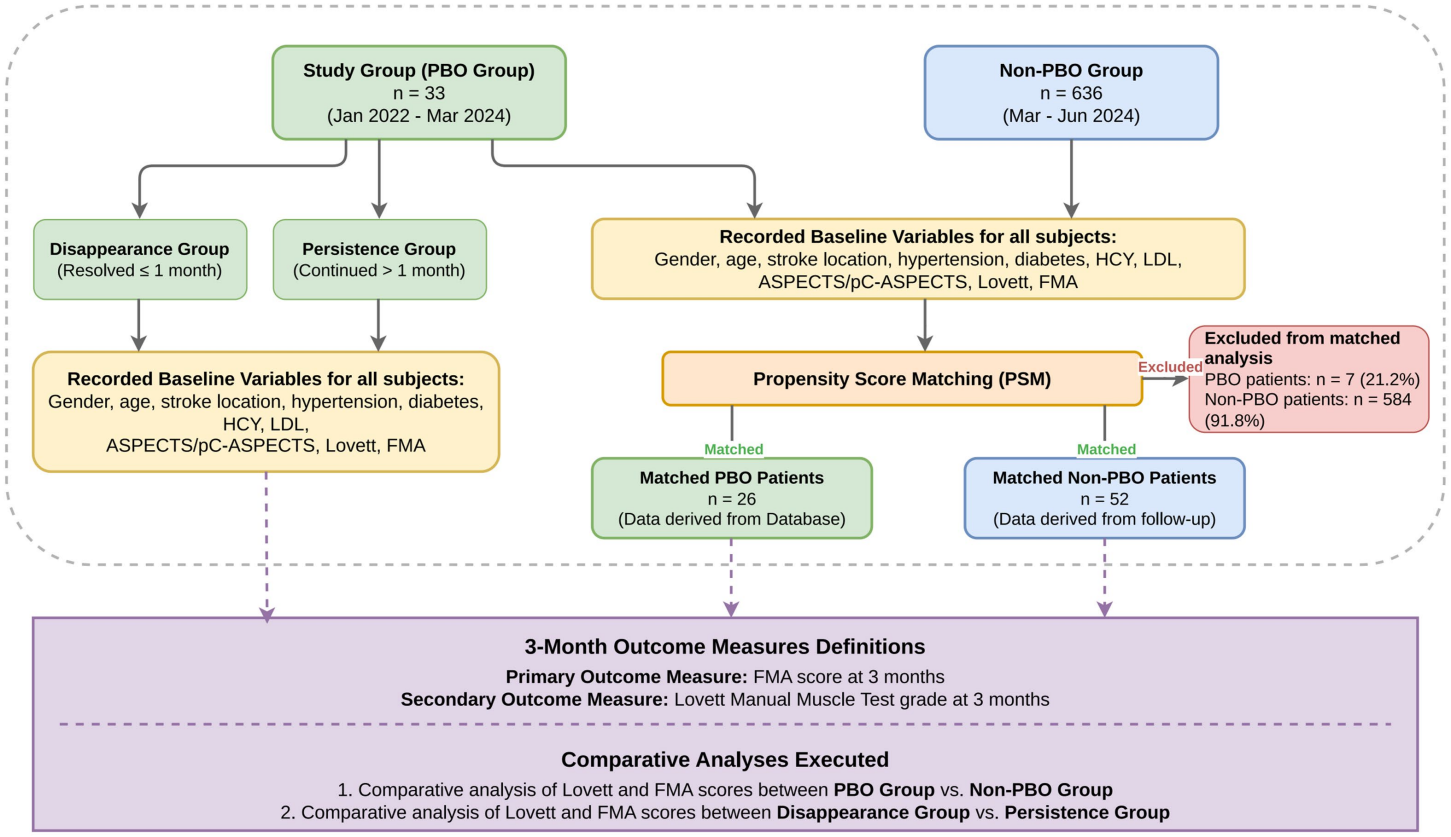
The participant flow diagram on post-stroke PBO and limb function recovery.

Since PBO is relatively rare, to ensure comparability in our study, we used PSM to select patients with comparable baseline characteristics from the group without PBO to serve as the non-PBO group. Because the initial severity of stroke is a key determinant of post-stroke prognosis, we accounted for baseline severity in our matching strategy. The National Institutes of Health Stroke Scale (NIHSS) covers both motor and non-motor domains (e.g., consciousness, language, sensory loss, and neglect). Since the primary objective of this study was to examine the association between PBO and motor recovery, and the outcome measures were motor-specific indicators (FMA score and Lovett score), we prioritized motor impairment indicators at admission in the propensity score model. Consequently, baseline FMA scores, Lovett scores, lesion location, and ASPECTS/pC-ASPECTS were included as surrogate measures of initial severity related to motor recovery (11). Another reason NIHSS was not included is that it is not fully available in retrospective registry databases. This study employed R 4.3.2 (2023-10-31 ucrt, x86_64-w64-mingw32/×64 64-bit) to construct the matching model. The MatchIt package (version 4.5.5) was used for 1:2 nearest-neighbor PSM without replacement, with a clamp value set at 0.02. The dependent variables were defined as the occurrence (PBO) versus non-occurrence (no PBO) of PBO. Independent variables included gender, age, hypertension, diabetes, hyperlipidemia, homocysteine levels, stroke location, ASPECT score, Lovett score, and FMA score. Following PSM, 26 PBO patients were successfully matched with 52 non-PBO patients. The remaining 7 PBO patients (21.2%) and 584 non-PBO patients (91.8%) were excluded from the matched analysis due to lack of suitable matches within the specified caliper (0.02). A participant flow diagram is presented in Figure 1.

### 3.6 Statistical analysis

Data analysis was performed using SPSS 23.0 statistical software. Continuous variables were first tested for normality using the Shapiro–Wilk test, and homogeneity of variances was assessed using Levene’s test. Variables that met both criteria (age, HCY, LDL, ASPECTS/pC-ASPECTS, and FMA) were expressed as mean ± standard deviation, and intergroup comparisons were performed using the independent samples *t*-test. Variables that did not meet either of the above criteria were expressed as the median and interquartile range, and comparisons between groups were performed using the Mann–Whitney U test. Ordinal variables (Lovett score) were expressed as the median and interquartile range and analyzed using the Mann–Whitney U test. Categorical variables (gender, hypertension, diabetes, and stroke location) were expressed as frequencies and percentages, and comparisons were performed using the chi-square test.

Given the exploratory nature of this study and the limited sample size, to avoid increasing the risk of Type II errors, no correction for multiple comparisons was performed. All tests were two-sided, and a *p*-value < 0.05 was considered statistically significant. All *p*-values are reported as nominal values, and results should be interpreted accordingly.

## 4 Result

### 4.1 Comparison of baseline data between the two patient groups before and after matching

Before PSM, there were substantial differences in baseline functional severity between the PBO and non-PBO groups. Specifically, the PBO group had significantly lower Lovett scores (median [IQR]: 2 [1, 2] vs. 3 [2, 4], *p* < 0.001) and lower FMA scores (13.82 ± 5.92 vs. 30.08 ± 13.94, *p* < 0.001) compared to the non-PBO group, this was due to the fact that some patients in the non-PBO group presented with non-motor symptoms. These variables, along with other baseline characteristics, were included in the propensity score model to ensure balanced comparisons after matching. After matching, no statistically significant differences were observed in baseline characteristics between the two groups (*p* > 0.05), as shown in Table 1.

**Table 1:** Comparison of baseline characteristics between the two patient groups before and after matching.

	Before matching					After matching				
Variable	PBO group (*n* = 33)	Non-PBO group (*n* = 636)	95%CI	*χ*^2^/*t*/*Z*	*p*	PBO group (*n* = 26)	Non-PBO group (*n* = 52)	95%CI	*χ*^2^/*t*/*Z*	*p*
Sex			0.56, 2.30	0.115	0.735			0.28, 2.10	0.271	0.603
Male	19 (57.6%)	384 (60.5%)				17 (65.4%)	37 (71.2%)			
Female	14 (42.4%)	251 (39.5%)				9 (34.6%)	15 (28.8%)			
Age	60.30 ± 6.92	59.86 ± 6.64	−0.89, 3.88	1.216	0.225	61.42 ± 6.57	58.96 ± 6.89	−0.81,5.74	1.497	0.138
HTN	29 (87.9%)	48 2(75.8%)	0.39, 1.87	0.159	0.690	19 (73.1%)	37 (71.2%)	0.32, 2.61	0.032	0.859
DM	13 (39.4%)	230 (36.2%)	0.56, 2.35	0.142	0.707	11 (42.3%)	17 (32.7%)	0.25, 1.75	0.696	0.404
HCY (μmol/L)	17.38 ± 2.14	16.88 ± 2.09	−0.234, 1.23	1.336	0.182	17.22 ± 2.17	17.18 ± 1.78	−0.88, 0.96	0.092	0.927
LDL (mmol/L)	2.40 ± 0.68	2.24 ± 0.67	−0.07, 0.39	1.350	0.177	2.35 ± 0.69	2.34 ± 0.57	−0.28, 0.31	0.095	0.924
Stroke location			0.25,1.26	2.042	0.153			0.18,1.58	1.300	0.254
Anterior circulation	25 (75.8%)	404 (63.5%)				18 (69.2%)	42 (80.8%)			
Posterior circulation	8 (24.2%)	232 (36.5%)				8 (30.8%)	10 (19.2%)			
ASPECT	6.61 ± 1.09	6.89 ± 1.56	−0.83,0.25	−1.039	0.299	6.54 ± 0.99	6.12 ± 1.23	−0.13,0.98	1.522	0.132
Lovett M (P25, P75)	2 (1, 2)	3 (2, 4)	NA	−6.966	<0.001	2 (1, 2)	1 (1, 2)	NA	−1.020	0.308
FMA	13.82 ± 5.92	30.08 ± 13.94	−21.05,-11.47	−6.667	<0.001	14.58 ± 5.15	14.60 ± 4.77	−2.36, 2.32	−0.016	0.987

HTN, Hypertension; DM, Diabetes mellitus; HCY, Homocysteine; LDL, Low-density lipoprotein; ASPECT, Alberta Stroke Program Early CT Score; Lovett: Lovett Muscle Strength Grading Scale; FMA: Fugl-Meyer Assessment Scale.

### 4.2 Comparison of Lovett and FMA scores between PBO and non-PBO groups after 3 months

**Figure 2:**
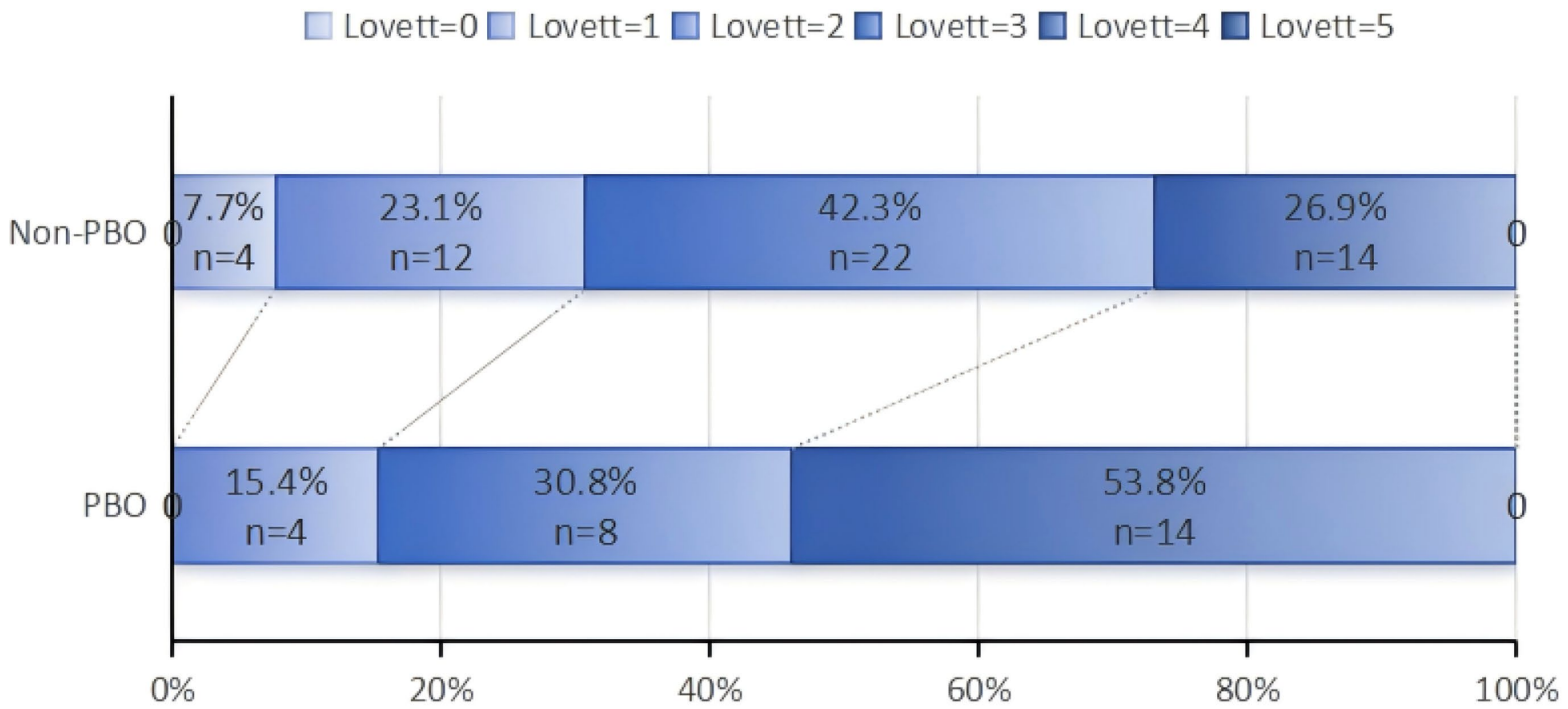
Comparison of Lovett scores between the PBO group and the non-PBO group at 3 months.

We evaluated the Lovett and FMA scores at 3 months in the matched PBO group (*n* = 26) and the non-PBO group (*n* = 52). After 3 months, the PBO group demonstrated significantly higher Lovett grades (median [IQR]:4 [3, 4] vs. 3 [2, 4], *p* < 0.05), as shown in Figure 2, and FMA scores (33.85 ± 7.29 vs. 30.08 ± 3.08, 95%CI [−0.567, 6.971], *p* < 0.05) compared to the non-PBO group, as shown in Table 2.

**Table 2:** Comparison of Lovett and FMA scores between PBO and Non-PBO groups at 3 months.

Variable	PBO group (*n* = 26)	Non-PBO group (*n* = 52)	95%CI	*t*/*Z*	*p*
LovettM (P25, P75)	4 (3, 4)	3 (2, 4)	NA	−2.362	0.018
FMA	33.85 ± 7.29	30.08 ± 3.08	−0.567,6.971	2.344	0.022

Lovett, Lovett Muscle Strength Grading Scale; FMA, Fugl-Meyer Assessment Scale.

### 4.3 Comparison of baseline data between the PBO persistence group and the PBO disappearance group

Based on whether PBO persisted for more than 1 month or resolved, we divided the PBO group (*n* = 33) into a PBO Persistence Group (*n* = 14) and a PBO Disappearance Group (*n* = 19), and compared the baseline data between the two groups. No statistically significant differences were observed in baseline characteristics between the two groups (*p* > 0.05), as shown in Table 3.

**Table 3:** Comparison of baseline data between the PBO persistence group and the PBO disappearance group.

Variable	PBO persistence group (*n* = 14)	PBO disappearance group (*n* = 19)	95%CI	*χ*^2^/*t*/*Z*	*p*
Sex (Male/female)			0.144, 2.371	0.571	0.450
Male	7 (50%)	12(63.2%)			
Female	7 (50%)	7,(6.8%)			
Age	63.71 ± 6.41	59.53 ± 6.89	−0.621,8.998	1.776	0.086
HTN	12 (85.7%)	13(68.4%)	0.061,2.146	1.313	0.252
DM	7 (50%)	6(31.6%)	0.111,1.921	1.146	0.284
HCY (μmol/L)	17.39 ± 2.30	17.36 ± 2.08	−1.531,1.591	0.039	0.969
LDL (mmol/L)	2.41 ± 0.74	2.40 ± 0.66	−0.486,0.510	0.050	0.960
Stroke location			0.255,6.715	0.105	0.746
Anterior circulation	12 (78.6%)	14 (73.7%)			
Posterior circulation	3 (21.4%)	5 (26.3%)			
ASPECT	6.71 ± 1.27	6.53 ± 0.96	−0.603, 0.979	0.485	0.631
Lovett	1 (0, 2)	1 (2, 2)	NA	−1.284	0.199
FMA	11.93 ± 1.54	15.11 ± 5.01	−7.274, 0.921	−1.581	0.124

HTN, Hypertension; DM, Diabetes mellitus; HCY, Homocysteine; LDL, Low-density lipoprotein; ASPECT, Alberta Stroke Program Early CT Score; Lovett, Lovett Muscle Strength Grading Scale; FMA, Fugl-Meyer Assessment Scale.

### 4.4 Comparison of Lovett and FMA scores between the PBO persistence group and the PBO disappearance group at 3 months

After 3 months, the PBO Disappearance Group demonstrated significantly higher FMA scores than the PBO Persistence Group (27.00 ± 8.59 vs. 34.11 ± 8.04, 95%CI[−13.101, −1.062], *p* < 0.05). No statistically significant difference was observed between the two Lovett grades (median [IQR]: 3 [2, 4] vs. 4 [3, 4], *p* > 0.05), as shown in Table 4.

**Table 4:** Comparison of Lovett grading and FMA Scores at 3 months between the PBO persistence group and the PBO discontinuation group.

Variable	PBO persistence group (*n* = 14)	PBO disappearance group (*n* = 19)	95%CI	*t*/*Z*	*p*
LovettM (P25, P75)	3 (2, 4)	4 (3, 4)	NA	−1.594	0.111
FMA	27.00 ± 8.59	34.11 ± 8.04	−13.101, −1.062	−2.437	0.021

Lovett, Lovett Muscle Strength Grading Scale; FMA, Fugl-Meyer Assessment Scale.

## 5 Discussion

PBO represents a distinct and often overlooked clinical manifestation in stroke patients with hemiplegia, characterized by involuntary elevation of the paralyzed limb during yawning (12). Previous studies have primarily consisted of a small number of case reports; as shown in Table 5, these reports have proposed possible pathophysiological mechanisms, but no studies have specifically examined the relationship between this phenomenon and functional outcomes (13–16). Among the 33 patients exhibiting PBO in this study, 31 demonstrated involuntary elevation solely in the upper limb, while 2 exhibited simultaneous elevation in both upper and lower limbs. Given this phenomenon, the study analyzed only upper limb function using the Lovett classification and FMA score. The Lovett classification is frequently used clinically for its intuitive assessment of limb functional changes. The FMA score quantifies upper limb motor ability through various movements, providing more accurate evaluation of fine motor skills (17). Yawning is a complex physiological behavior involving multi-level neural regulation: its initiation and control primarily rely on the paraventricular nucleus of the hypothalamus, which projects signals via oxytocinergic, cholinergic, and other neuronal groups to the hippocampus, brainstem (pons, medulla), and even the spinal cord (18). Pathological or excessive yawning represents a clinically significant manifestation following acute stroke. Research indicates it frequently signals damage to the brainstem reticular formation or cortical/subcortical regions (particularly the insula and caudate nucleus). This demonstrates direct overlap between the neural pathways governing yawning and the lesion site in stroke (19).

**Table 5:** Literature reports on the PBO phenomenon.

References	Design	Sample size	Key Findings
Töpper et al. (6)	Case Report	*n* = 3	Involuntary stretching during yawning in patients with pyramidal tract lesions.
Walusinski et al. (4)	Case Report	*n* = 4	Clarifies the definition of PBO and proposes a mechanism for the release of cortical inhibition.
Walusinski et al. (23)	Case Report	*n* = 6	It has been proposed that the lateral reticular nucleus and the spinocerebellar tract are involved in the development of PBO.
De Lima et al. (21)	Case Report	*n* = 3	This was the first time the PBO phenomenon, manifested as lower limb movements, was described.
Jung et al. (13)	Case Report	*n* = 1	It has been proposed that the “emotional motor system” may be involved in the development of PBO.
Wu et al. (14)	Case Report	*n* = 1	Crossed cerebellar diaschisis and PBO might share similar neuroanatomical pathways and be valuable for predicting clinical recovery after stroke.
Sanjith Aaron et al. (16)	Case Report	*n* = 1	A patient with a pontine infarction exhibited the PBO phenomenon.
Lim et al. (5)	Case Report	*n* = 1	Proprioceptive loop theory and Emotional motor system.
Chowdhury et al. (15)	Case Report	*n* = 1	Disinhibition of subcortical structures by cortical damage that may release the reticular brainstem formation interconnected with motor pathways and may be activated by stimuli such as yawning.

**Figure 3:**
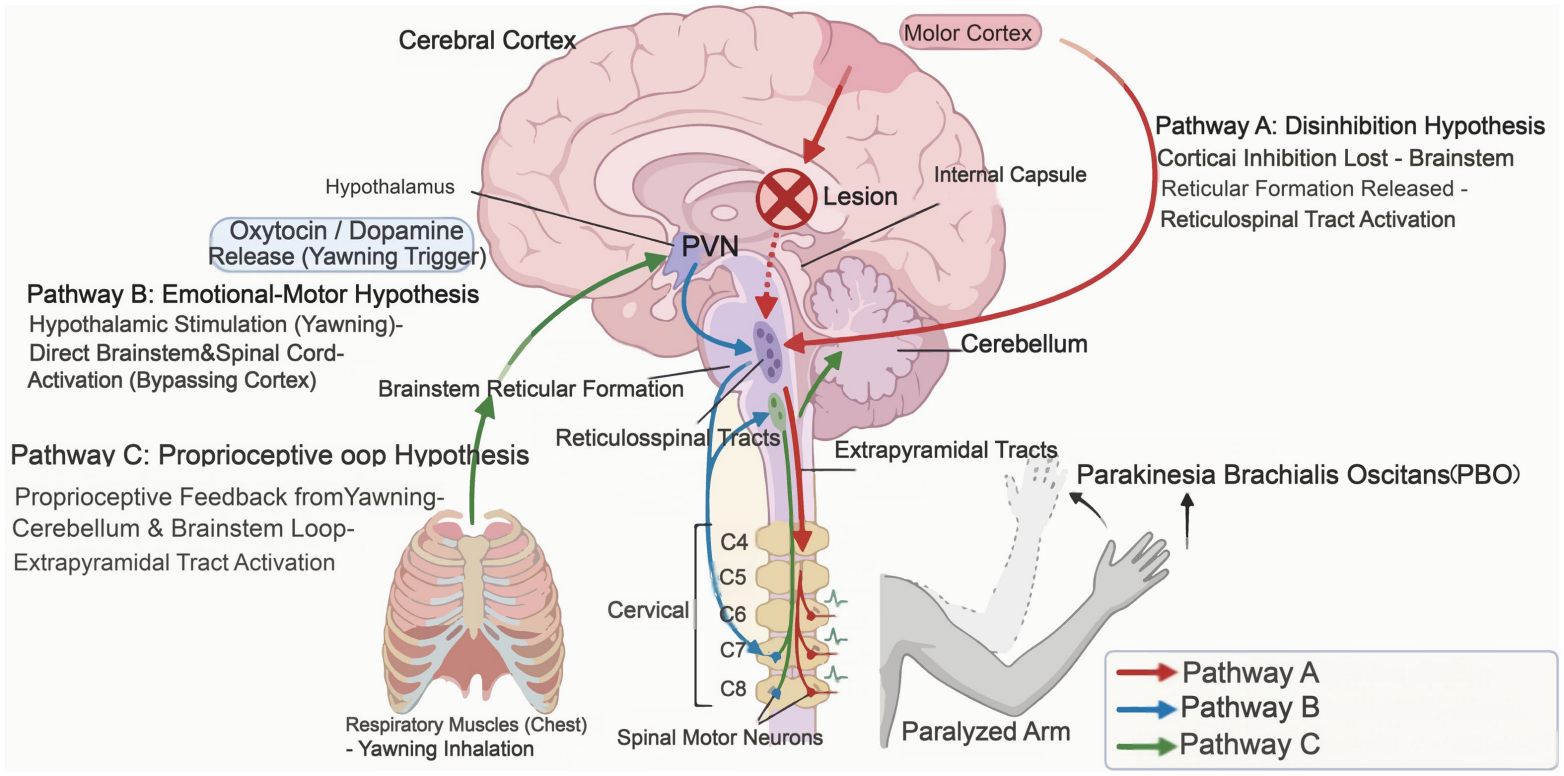
Neurological pathways of Parakinesia brachialis oscitans (PBO) in post-stroke patients.

Due to the relatively low incidence of PBO, this study employed PSM to select patients with comparable baseline characteristics for comparison with the PBO cohort, ensuring research rigor (20). A total of 33 PBO patients were recorded, matched 1:2 via PSM, resulting in 26 patients (PBO group) matched to 52 corresponding patients (non-PBO group). Further analysis revealed that at 3 months, the PBO group demonstrated statistically significant superiority over the non-PBO group in Lovett muscle strength grading and FMA scores (*p* < 0.05). In previous case reports, several neurophysiological hypotheses have been proposed, including three main hypotheses: the disinhibition of cortical-to-subcortical motor pathways, the activation of the extrapyramidal motor-emotional system, and proprioceptive feedback transmitted via the cerebellar-brainstem-spinal cord circuit; all of these may contribute to the occurrence of involuntary limb movements during yawning, as shown in Figure 3 (21–23). However, these mechanisms were not directly tested in the present study. Our findings are primarily interpreted at the clinical association level, rather than as mechanistic evidence. Regardless of the hypothesis, the occurrence of PBO suggests that primitive motor networks at the brainstem and spinal cord level remain relatively intact or exhibit heightened excitability. These networks include the reticulospinal system and cerebellar-brainstem connections, and their preservation occurs despite disruption of the corticospinal tract, which serves as the primary descending motor pathway. In this study, patients in the PBO group demonstrated significantly superior muscle strength and motor function scores at 3 months compared to the non-PBO group, suggesting that PBO may represent an observable clinical sign associated with the integrity and excitability of primitive motor pathways. The PBO phenomenon is compatible with the hypothesis that the lower-level motor centers in the brainstem and spinal cord retain functional integration capabilities and that neural connections to limb muscles remain partially intact. The ability to perform the complex coordinated movement of PBO may indicate that the “alternative pathway” from the brainstem to the anterior horn cells of the spinal cord is structurally preserved and functionally activatable. Compared to non-PBO patients, those exhibiting PBO possess more complete “neurological reserve.” During the process of neural reorganization following a stroke, these preserved and activatable primitive pathways provide a potential anatomical and physiological basis for the recovery of motor function. Rehabilitation training may be able to leverage these pathways to compensate for the impaired function of the corticospinal tract, thereby achieving superior functional recovery outcomes.

Literature reports suggest that PBO may be associated with poor patient outcomes (12). We classified patients whose PBO resolved within 1 month post-stroke as the disappearance group and those with persistent PBO as the persistence group. Statistical analysis of Lovett muscle strength grades and FMA scores between the two groups after stroke revealed no statistically significant difference (*p* > 0.05). Three months later, the FMA scores in the disappearance group were significantly higher than those in the persistence group (*p* < 0.05). There was no statistically significant difference in Lovett muscle strength grading between the two groups (*p* > 0.05). In this study, the FMA score focuses more on evaluating isolated movements, coordination, and fine motor control, which are highly dependent on the integrity of the corticospinal tract (24). The Lovett scale provides a relatively coarse ordinal estimate of muscle strength, mainly reflecting the ability to move against gravity or resistance, and may be less sensitive to subtle improvements in selective motor control, coordination, and distal upper-limb performance (25). Compared to the FMA, the Lovett scale is less sensitive as a measure of physical fitness when assessing changes in athletic ability. The higher FMA scores in the disappearance group suggest better recovery of corticospinal tract function. Regarding this outcome, we venture the hypothesis that the persistent PBO phenomenon may serve as a clinical marker of the motor system’s failure to transition successfully from a “lower-level compensatory mode” to a “higher-level dominant mode” post-stroke. This may indicate that neural remodeling has become stuck in an inefficient plateau phase. The superior function in the disappearance group likely stems not only from the “re-inhibition” of lower-level centers following corticospinal tract recovery but also from an active, successful “functional hierarchical transition” process. In the persistent PBO group, impaired recovery of the corticospinal tract leaves lower pathways persistently overactive and “unleashed” due to lack of higher-level control. This state itself may hinder corticospinal reorganization through mechanisms like synaptic competition, creating a vicious cycle (26). Consequently, functional recovery remains stalled at a plateau, preventing further improvement.

The early disappearance of PBO may indicate successful remodeling of corticospinal tract function and the “return” of higher-level motor control, signaling that the nervous system is progressing toward a more optimal functional reorganization. However, at present, these observations should not be taken as evidence to modify rehabilitation intensity or timing in routine clinical practice. Rather, they suggest that PBO status may be worth evaluating as a stratification variable in future prospective or interventional rehabilitation studies.

In this PSM retrospective cohort, the presence of PBO after ischemic stroke was associated with better 3-month motor outcomes, as reflected by higher Lovett grades and FMA scores compared with matched patients without PBO. Among patients with PBO, early disappearance of the phenomenon within 1 month was associated with better 3-month FMA performance than persistent PBO. These findings suggest that PBO may be a clinically observable sign associated with short-term motor recovery trajectory after stroke.

Several methodological limitations should be considered. First, this was a retrospective single-center study, which may limit generalizability. Second, follow-up was limited to 3 months, and therefore longer-term recovery trajectories could not be evaluated. Third, although all patients received rehabilitation management, the content, intensity, frequency, and adherence of rehabilitation interventions during follow-up were not standardized or quantitatively captured, which may have influenced functional outcomes. Fourth, there were marked baseline differences in functional severity before matching, and baseline severity-related variables played an important role in the PSM process. While this approach improved comparability, it may also affect the interpretation of between-group outcome differences and reduce the representativeness of the matched sample relative to the source population.

Residual confounding cannot be excluded despite PSM, because matching can only address measured variables. Unmeasured or incompletely measured factors, such as detailed lesion burden, corticospinal tract integrity, acute treatment characteristics, and rehabilitation exposure, may still have influenced the observed associations, particularly given the modest sample size and limited subgroup numbers. This is a retrospective observational study design;the present study relied primarily on clinical functional scales without complementary objective biomarkers, such as lesion-specific imaging analyses, diffusion-based assessment of corticospinal tract integrity, or electrophysiological measures. Whether PBO can be considered a valid prognostic marker requires larger-scale, multicenter, prospective studies with longer follow-up periods and multimodal assessments of the underlying mechanisms. Future research should aim to employ multimodal neuroinformation fusion (synchronous acquisition and analysis of high-resolution MRI, electroencephalography, surface electromyography) to quantitatively analyze PBO-specific neural circuit characteristics.

## Data Availability

The data supporting the findings of this study are available in the National Population Health Data Center (NPHDC) at: https://www.ncmi.cn, with named [Yawning-induced motor miscoordination of the biceps brachii after stroke]. Access to the data may require registration and approval from the data provider.

## References

[1] Ma Q Li R Wang L Yin P Wang Y Yan C . Temporal trend and attributable risk factors of stroke burden in China, 1990-2019: an analysis for the global burden of disease study 2019. Lancet Public Health. (2021) 6:e897–906. doi: 10.1016/S2468-2667(21)00228-0, 34838196 PMC9047702

[2] Nave AH Rackoll T Grittner U Bläsing H Gorsler A Nabavi DG . Physical fitness training in patients with subacute stroke (PHYS-STROKE): multicentre, randomised controlled, endpoint blinded trial. *BMJ (Clin res ed)*. (2019) 366:l5101. doi: 10.1136/bmj.l5101, 31533934 PMC6749174

[3] Tu W-J Hua Y Yan F Bian H Yang Y Lou M . Prevalence of stroke in China, 2013-2019: a population-based study. Lancet Reg Health, West Pac. (2022) 28:100550. doi: 10.1016/j.lanwpc.2022.100550, 36507089 PMC9727498

[4] Walusinski O Quoirin E Neau J-P. Parakinesia brachialis oscitans. Rev Neurol (Paris). (2005) 161:193–200. doi: 10.1016/s0035-3787(05)85022-2, 15798518

[5] Lim ICZY Neo S. Parakinesia brachialis oscitans: old sign, new findings. Stroke. (2022) 53:e60–2. doi: 10.1161/STROKEAHA.121.037124, 34937420

[6] Töpper R Mull M Nacimiento W. Involuntary stretching during yawning in patients with pyramidal tract lesions: further evidence for the existence of an independent emotional motor system. Eur J Neurol. (2003) 10:495–9. doi: 10.1046/j.1468-1331.2003.00599.x, 12940828

[7] Kleindorfer DO Towfighi A Chaturvedi S Cockroft KM Gutierrez J Lombardi-Hill D . 2021 guideline for the prevention of stroke in patients with stroke and transient ischemic attack: a guideline from the american heart association/american stroke association. Stroke. (2021) 52:e364–467. doi: 10.1161/STR.0000000000000375, 34024117

[8] Naganuma M Tachibana A Fuchigami T Akahori S Okumura S Yi K . Alberta stroke program early CT score calculation using the deep learning-based brain hemisphere comparison algorithm. J Stroke Cerebrovasc Dis: Off J Natl Stroke Assoc. (2021) 30:105791. doi: 10.1016/j.jstrokecerebrovasdis.2021.105791, 33878549

[9] Kniep HC Elsayed S Nawabi J Broocks G Meyer L Bechstein M . Imaging-based outcome prediction in posterior circulation stroke. J Neurol. (2022) 269:3800–9. doi: 10.1007/s00415-022-11010-4, 35257203 PMC9217773

[10] Wang Z Zhang T Fan J Gu F Yu Q Wang H . Clinical validation of automated depth camera-based measurement of the Fugl-Meyer assessment for upper extremity. Clin Rehabil. (2024) 38:1091–100. doi: 10.1177/02692155241251434, 38693881

[11] Pellicciari L Sodero A Campagnini S Guolo E Basagni B Castagnoli C . Factors influencing trunk control recovery after intensive rehabilitation in post-stroke patients: a multicentre prospective study. Top Stroke Rehabil. (2023) 30:109–18. doi: 10.1080/10749357.2021.2016099, 34994302

[12] Chowdhury A Datta AK Biswas S Biswas A. Parakinesia brachialis oscitans - a rare post-stroke phenomenon. Tremor Other Hyperkinetic Mov (N Y NY). (2022) 12:6. doi: 10.5334/tohm.680, 35433110 PMC8916051

[13] Jung N-Y Ahn B-Y Park K-H Chung C-S Na DL Kim E-J. A case of parakinesia brachialis oscitans. Clin Neurol Neurosurg (2012) 114:156–158. doi: 10.1016/j.clineuro.2011.08.020 21955580

[14] Wu Y-T Chang S-T Chen L-C Li T-Y. Concurrence of crossed cerebellar diaschisis and parakinesia brachialis oscitans in a patient with hemorrhagic stroke. Case Rep Med (2013) 2013:519808. doi: 10.1155/2013/519808 24307905 PMC3836471

[15] Chowdhury A Datta AK Biswas S Biswas A. Parakinesia brachialis oscitans - a rare post-stroke phenomenon. Tremor Other Hyperkinetic Mov (2022) 12:6. doi: 10.5334/tohm.680 PMC8916051 35433110

[16] Aaron S Al Hashmi A. Parakinesia brachialis oscitans due to brain stem infarct. Dubai Med J (2019) 2:20–22. doi: 10.1159/000500497

[17] Wiesner K Schwarz A Meya L Kaufmann JE Traenka C Luft AR . Interrater reliability of the fugl-meyer motor assessment in stroke patients: a quality management project within the ESTREL study. Front Neurol. (2024) 15:1335375. doi: 10.3389/fneur.2024.1335375, 38651097 PMC11034517

[18] Wani PD Agarwal M. The science of yawning: exploring its physiology, evolutionary role, and behavioral impact. J Fam Med Prim Care. (2025) 14:3115–20. doi: 10.4103/jfmpc.jfmpc_1677_24, 41041241 PMC12488162

[19] Teive HAG Munhoz RP Camargo CHF Walusinski O. Yawning in neurology: a review. Arq Neuropsiquiatr. (2018) 76:473–80. doi: 10.1590/0004-282X20180057, 30066799

[20] Austin PC. Optimal caliper widths for propensity-score matching when estimating differences in means and differences in proportions in observational studies. Pharm Stat. (2011) 10:150–61. doi: 10.1002/pst.433, 20925139 PMC3120982

[21] de Lima PMG Munhoz RP Becker N Teive HAG. Parakinesia brachialis oscitans: report of three cases. Park Relat Disord. (2012) 18:204–6. doi: 10.1016/j.parkreldis.2011.09.020, 22018911

[22] Moritz C Field-Fote EC Tefertiller C van Nes I Trumbower R Kalsi-Ryan S . Non-invasive spinal cord electrical stimulation for arm and hand function in chronic tetraplegia: a safety and efficacy trial. Nat Med. (2024) 30:1276–83. doi: 10.1038/s41591-024-02940-9 38769431 PMC11108781

[23] Walusinski O Neau J-P Bogousslavsky J. Hand up! Yawn and raise your arm. Int J Stroke. (2010) 5:21–7. doi: 10.1111/j.1747-4949.2009.00394.x, 20088989

[24] Rowe V Blanton S Aycock D Hayat MJ Ali SZ. Remote delivery of the fugl-meyer assessment for the upper extremity: a pilot study to assess feasibility, reliability, and validity. Arch Rehabil Res Clin Transl. (2023) 5:100261. doi: 10.1016/j.arrct.2023.100261, 37312985 PMC10258373

[25] Ren Z Ye S Nie Q Feng J Liu K Li Q . Application of digitization and visualization-based muscle strength measurement in ischemic stroke patients with motor dysfunction. Sci Rep. (2023) 13:17507. doi: 10.1038/s41598-023-44826-9, 37845368 PMC10579306

[26] Mason J Frazer A Horvath DM Pearce AJ Avela J Howatson G . Adaptations in corticospinal excitability and inhibition are not spatially confined to the agonist muscle following strength training. Eur J Appl Physiol. (2017) 117:1359–71. doi: 10.1007/s00421-017-3624-y 28455814

